# Fecal human β-defensin-2 (hBD-2) levels and gut microbiota patterns in preterm neonates with different feeding patterns

**Published:** 2019-04

**Authors:** Brigitta I.R.V. Corebima, Rinawati Rohsiswatmo, Pramita Gayatri, Sanjay Patole

**Affiliations:** 1 Department of Neonatology, Child Health Division, Faculty of Medicine Brawijaya University, Dr. Saiful Anwar General Hospital, Malang, Indonesia; 2 Department of Neonatology, Child Health Division, Faculty of Medicine University of Indonesia, Dr. Cipto Mangunkusumo General Hospital, Jakarta, Indonesia; 3 Department of Gastrohepatology, Child Health Division, Faculty of Medicine University of Indonesia, Dr. Cipto Mangunkusumo General Hospital, Jakarta, Indonesia; 4 Department of Neonatal Paediatrics, King Edward Memorial Hospital for Women, Perth, Australia

**Keywords:** Human B-defensin-2, Intestinal microbiota pattern, Preterm neonates

## Abstract

**Background and Objectives:**

Human β-defensin-2 (hBD-2) is an essential antibacterial peptide involved in innate immunity and is expressed in breast milk and intestinal mucosa. The aim of this study was to investigate fecal hBD-2 levels and gut microbiota in preterm neonates with different feeding patterns.

**Materials and Methods:**

This study was cross-sectionally designed and included 44 preterm neonates categorized into four groups as follows: breast milk only, breast milk predominant, formula milk predominant, formula milk only. The study was conducted at the Neonatology Ward, National Center Hospital Cipto Mangunkusumo, Jakarta from November 2016 to April 2017. hBD-2 levels were measured by ELISA. Intestinal bacteria were quantified by qPCR.

**Results:**

hBD-2 levels were significantly different between groups (one-way ANOVA, p=0.004) and the highest value of hBD-2 was found in the formula milk predominant group (344.87±61.2 ng/mL). hBD-2 levels were positively correlated with feeding pattern (Spearman correlation test, p=0.009, r=0.391). There were no significant differences in the total number of specific intestinal microbiota (*Bifidobacterium, Lactobacillus* and *Klebsiella*) among groups (one-way ANOVA, p>0.05). Interestingly, the formula milk only group had the highest amount of *Klebsiella* compared with other groups. hBD-2 levels were not correlated with the quantity of *Bifidobacterium, Lactobacillus* and *Klebsiella* (Pearson correlation test, p>0.05).

**Conclusion:**

hBD-2 levels were significantly higher in the formula milk predominant group compared with the breast milk only group. Gut microbiota patterns showed that *Bifidobacterium* and *Lactobacillus* were higher in the breast milk only group, while *Klebsiella* was higher in formula milk group, although this difference was not statistically significant.

## INTRODUCTION

Preterm birth is defined as birth prior to 37 completed gestational weeks (1) and global epidemiological data show that approximately 11% of births are annually classified as premature (2). Problems related to preterm birth are mainly caused by immature organ function and severe infection (3–4). Severe infection, such as sepsis, pneumonia, and necrotizing enterocolitis, contributes to the mortality of preterm neonates worldwide, and is associated with immature innate and adaptive immune responses (5–6).

The innate immune response in the intestine is mediated by commensal or non-pathogenic microbiota (7) and antimicrobial peptides, including human β-defensin 2 (hBD-2) (8). Inappropriate regulation of this peptide can lead to intestinal disorders initiated by inflammation of the gastrointestinal tract. Gastrointestinal inflammation is a known pitfall in the management of preterm neonates and can result in decreases in the amount of feeding or feeding tolerance, and even lead to more serious conditions (9–10). The gastrointestinal system in preterm neonates is vulnerable to colonization by pathogenic bacteria, while prolonged antibiotics use and hospitalization can affect the intestinal microbiome, leading, in turn, to inflammation.

Breast milk can help to improve and maintain a normal composition of intestinal bacteria, and also alter the characteristics of the intestinal microbiome from pathogenic to protective. Breast milk comprises of high levels of hBD2, *Lactobacillus*, and *Bifidobacterium*, which has a protective effect toward inflammation (11–13). Nutritional administration in neonates is problematic because not all preterm neonates can be fed with breast milk exclusively.

The aim of this study was to identify the baseline characteristics (i.e. gestational age, birth weight, history of asphyxia, hyaline membrane disease, cardiovascular disorders) of preterm neonates administered 4 different feeding patterns (i.e. breast milk, breast milk predominant, formula predominant) as well as the characteristics of the mothers of the preterm neonates (intrauterine infection, hypertension, asthma, preeclampsia). Correlations between the prevalence of gastrointestinal inflammation and fecal hBD-2 levels with other inflammatory markers (CRP, IT ratio) were inferred and comparisons of normal flora patterns in preterm neonates (gestational age 28–34 weeks) with different feeding patterns (breast milk, breast milk predominant, formula milk predominant, formula milk only) were made.

## MATERIALS AND METHODS

### Study design.

This study was designed as a cross sectional study to investigate the association of fecal human defensin levels and patterns in the intestinal microbiota of preterm neonates (gestational age 28–34 weeks) provided with either breast milk only, breast milk predominantly, formula milk predominantly, or formula milk only. This study was conducted in the Neonatology Ward of the National Center Hospital Cipto Mangunkusumo, Jakarta from November 2016 until April 2017. This study was approved by the Medical/Health Research, Ethical Committee of Medical Faculty, Indonesia University/Cipto Mangunkusumo Hospital based on recommendation letter number 753/H2.F1/ETIK/2016.

### Study population.

The target population of this study was preterm neonates with gestational age 28–34 weeks. The accessible population of this study were preterm neonates who were admitted to the Neonatology Ward of National Center Hospital Cipto Mangunkusumo from September to December 2016. The inclusion criteria for subjects were: preterm neonates (gestational ages 28–34 weeks) admitted to National Center Hospital Cipto Mangunkusumo; mode of labor either vaginal delivery or abdominal delivery; fed with breast milk only, breast milk predominantly, formula milk predominantly, or formula milk only; family of patients agreed for child to be included in the research after explanation (i.e., informed consent). Exclusion criteria for this study were: preterm neonates with gastrointestinal disorder requiring surgical intervention, family of patients did not agree for child to be included in this research after explanation (i.e., informed consent).

A total of 44 subjects were divided into 4 groups: 11 subjects in the breast milk only group, 11 subjects in the breast milk predominant group, 13 subjects in the formula milk predominant group, and 9 subjects in the formula milk only group. Patients meeting inclusion criteria were included as research subjects, and patients meeting exclusion criteria were excluded as subjects. Parents or guardians were informed of inclusion and asked for written consent. After the selection process, all included subjects were documented for complete identity, working diagnosis at admission, and feeding group.

### Measurement of CRP and I/T Ratio.

All blood samples were drawn within 12 h of hospital admission. The C-reactive protein (CRP) level of subjects was measured using an immunoturbidimetric assay (Beckman, Carlsbad, CA 92010, USA) (14). Immature to total neutrophil (I/T) ratio was calculated from complete blood counts measured on hematology analyzer. All of laboratory procedures, including CRP, plateletcrit, and I/T ratio measurements, were conducted at the Laboratory of Clinical Pathology, Cipto Mangunkusumo Central Hospital, Indonesia. These data were obtained from medical records.

### Extraction of DNA from fecal samples.

Fecal samples were obtained from all subjects at the time of admission. DNA extraction was conducted as previously described (15). Briefly, 1 g of fecal sample was suspended in a solution containing 10 mL of saline and homogenized by vortexing for 1 min. The slurry was filtered through sterile gauze to remove any large particles and debris. A 1 mL aliquot of filtered fecal solution was centrifuged at 15,000 rpm for 2 min. The supernatants were discarded, and the pellets were treated with 20 μl of 6 mg/mL lysozyme and 30 μl of 50 U/mL mutanolysin at 37°C for 2 h, then with 0.1 mL of 10% sodium dodecyl sulfate and 80 μl of benzyl chloride at 60°C for 2 h, and finally with 80 μl of chloroform. After centrifugation at 12,500 rpm for 2 min, the supernatants were collected and DNA was obtained by alcohol precipitation. The precipitated DNA was suspended in 50 μl of deionized water and stored at −80°C until use. DNA extraction was performed at the Laboratory of Biomolecular Gastrohepatology, Faculty of Medicine, University of Indonesia.

### Quantitative Real-Time PCR.

The primers and probes for the 5′ nuclease assays were based on sequences of the 16S-23S intergenic spacer regions of different *Bifidobacterium* species, *Lactobacillus* species, and *Klebsiella* species according to previous studies (16–18). The oligonucleotides were adapted for qPCR suitability from previously published specific primers or probes using the Primer Express Software (Applied Biosystems, CA, USA). The specificity of the adapted primers was verified using the basic local alignment search tool (BLAST) to query the NCBI database using a nucleotide-nucleotide similarity search, and the absence of amplification of human DNA was tested empirically by PCR using DNA extracted from blood. Standard curves were constructed using PCR products of the 16S rRNA gene of *Bifidobacterium* species, *Lactobacillus* species, and *Klebsiella* species.

qPCR was conducted as previously described (19). Briefly, qPCR was performed on an ABI 7900 HT Sequence Detection System (PE Biosystems, Warrington, UK) using optical grade 96-well plates. The qPCR reaction was performed in a total volume of 25 μl using the SYBR Green PCR Core Reagents kit (PE Biosystems). Each reaction included 2.5 μl 10× SYBR Green buffer, 3 μl MgCl (25 mM), 2 μl dNTPs (2.5 mM), 0.25 μl AmpErase UNG (1 U/μl), 0.125 μl AmpliTaq Gold (5 U/μl), 1 μl of each primer (12.5 μM) and 2 μl of DNA samples (diluted 1/10). The reaction conditions for amplification of DNA were 50°C for 2 min, 95°C for 10 min, 40 cycles of 95°C for 15 s and 60°C for 1 min. Primer specificity was evaluated by melt-curve analysis of products after the last amplification cycle. qPCR was performed at the Laboratory of Biomolecular Gastrohepatology, Faculty of Medicine, University of Indonesia.

### Measurement of hBD-2.

Fecal hBD-2 was measured using a specific enzyme-linked immune assay (ELISA) according to the manufacturer's instructions (β–defensin 2; Immunodiagnostic AG, Bensheim, Germany; Cat: K6500, Lot: K6500-161025). The assay was conducted after all samples had been collected. The interval calibration of standards was 0.1–3.0 ng/mL, the detection limit of the assay was 0.03 mg/mL, and samples were diluted 200× prior to analysis. Values were extrapolated for samples with ELISA results outside the range of the calibration standard. Measurement of fecal hBD-2 was performed using a Bio-Rad model 680 Microplate Reader and the Microplate Manager software version 5.2.1 (Bio-Rad Laboratories, Inc., CA, USA).

## RESULTS

### Baseline characteristics.

The 44 subjects included in this study were classified into 4 groups based on feeding patterns: breast milk only, breast milk predominant, formula milk predominant, and formula milk only. Subject characteristics are detailed in Table 1. Statistical analyses found no significant differences among groups based on sex, age, gestational age, birth weight, body length, head circumference, mode of labor, body temperature, or mothers’ characteristics (incidence of maternal infection and severe pre-eclampsia). However, there were significant differences in neonates based on the characteristics of ventilator utilization, incidence of patent ductus arteriosus (PDA), and the usage of breast milk donor. Ventilator utilization and the incidence of patient ductus arteriosus were significantly higher in breast milk only groups, but the usage of a breast milk donor was significantly higher in the formula milk predominant group. The remaining neonate characteristics (incidence of asphyxia, sepsis, and oral hygiene problem) were not different among groups.

**Table 1.** Baseline characteristics of study groups

**Characteristics**		**Breast milk Only (n = 11)**	**Breast milk Predominant (n = 11)**	**Formula Milk Predominant (n = 12)**	**Formula Milk Only (n = 10)**	**p-value**
Sex (n)						
•	Male	2	7	6	6	0.133^a^
•	Female	9	4	6	4	
Age (day)		14	13.5±0.45	14.1±0.08	14	0.301^b^
Gestational age (week)		31.8±0.73	31.9±0.31	31.8±0.41	31.2±0.59	0.651^b^
Birth weight (gram)		1419.1±131.9	1699.5±62.6	1453.8±88.3	1498.2±139.4	0.237^c^
Body length (cm)		39.8±1.5	41.7±0.6	39.4±1.3	40.4±1.4	0.596^c^
Head circumference (cm)		29.0±0.4	30.1±0.4	29.5±0.8	29.1±0.7	0.534^c^
Body temperature (°C)		36.9±0.05	36.9±0.04	37.0±0.07	36.9±0.06	0.416^b^
Mode of Labor						
•	Abdominal delivery (n)	8	6	10	6	0.453^a^
•	Vaginal delivery (n)	3	5	2	4	
Mother's characteristics						
•	Maternal infection (n)	6	6	6	6	0.974^a^
•	Severe preeclampsia (n)	2	2	4	0	0.254^a^
Neonate's characteristics						
•	Asphyxia (n)	2	3	2	0	0.391^a^
•	Sepsis (n)	9	5	6	7	0.253^a^
•	Ventilator (n)	6	2	0	2	0.018^a^
•	PDA (n)	4	0	0	1	0.020^a^
•	Oral hygiene problem (n)	0	3	3	1	0.246^a^
•	Breast milk donor (n)	0	3	8	0	0.000^a^

Data presented as number of cases for nominal variables and mean ± SEM for numeric variables.

a p-value was determined by the Chi-square test;

b p-value was determined by the Kruskal-Wallis test;

c p-value was determined by one-way ANOVA.

Significant difference were defined as p < 0.05. PDA: patent ductus arteriosus

Table 2 shows the levels of various inflammatory markers, such as CRP and I/T ratio, as well as incidence of necrotizing enterocolitis in each group. Statistical analyses found no significant differences CRP levels or I/T ratio among all groups. No significant difference in the incidence of necrotizing enterocolitis was found among groups.

**Table 2.** Level of pro-inflammatory markers and the incidence of necrotizing enterocolitis

**Variables**	**Breast milk only (n = 11)**	**Breast milk predominant (n = 11)**	**Formula milk predominant (n = 12)**	**Formula milk only (n = 10)**	**p-value**
I/T ratio (×10^3^)	110.0±20.7	146.4±22.3	116.7±18.4	115.6±33.3	0.570^a^
CRP (ng/mL)	13.45±10.91	14.27±13.82	1.90±0.91	0.46±0.17	0.644^a^
Incidence of NEC (n)	3	0	2	1	0.298^b^

Data presented as number of cases for nominal variables and mean ± SEM for numeric variables.

a p-value was determined by the Kruskal-Wallis test;

b p-value was determined by the Chi-square test.

Significant difference were defined as p < 0.05. I/T ratio: immature to total neutrophil ratio; CRP: C-reactive protein; NEC: necrotizing enterocolitis

### hBD-2.

**Fig. 1.**
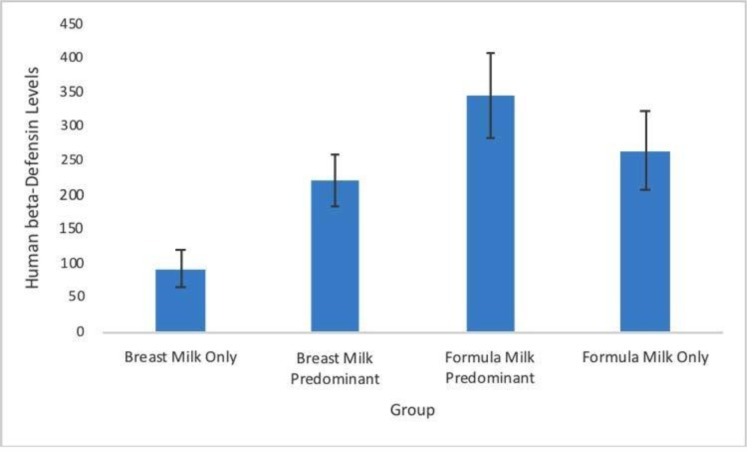
Human β-defensin 2 levels in each group. Data presented as mean±SEM (ng/mL). ^a^ p < 0.05 as compared to breast milk only group

Statistical analyses of hBD-2 levels in each group (Fig. 1) showed significant differences among groups (one-way ANOVA, p = 0.04). Moreover, post hoc analysis showed that hBD-2 levels in the formula milk predominant group (344.87±61.2 ng/mL) were significantly higher than in the breast milk only group (91.84±26.9 ng/mL, p = 0.02). However, post hoc analysis found no significant differences between hBD-2 levels in other groups (breast milk predominant: 221.52±37.6 ng/mL; formula milk only: 264.16±56.2 ng/mL). However, hBD-2 levels were significantly correlated with feeding pattern (Spearman correlation test, p = 0.009, r = 0.391).

### Intestinal microbiota pattern.

Table 3 shows the number of specific bacteria (i.e., *Bifidobacterium, Lactobacillus, Klebsiella*) and the ratio of each to total bacteria in each group. Statistical analyses showed no significant differences in the total number of *Bifidobacterium* (one-way ANOVA test, p = 0.561), *Lactobacillus* (one-way ANOVA, p = 0.327), and *Klebsiella* (one-way ANOVA, p = 0.134) among groups. Furthermore, there were also no significant differences of *Bifidobacterium* /total bacteria (one-way ANOVA, p = 0.739), *Lactobacillus* /total bacteria (one-way ANOVA, p = 0.267), or *Klebsiella* / total bacteria (one-way ANOVA, p = 0.342) among groups. Interestingly, the breast milk only group had the highest number of *Lactobacillus*. Moreover, *Bifidobacterium* count per total bacteria was higher in breast milk and breast milk predominant group. *Klebsiella* counts were highest in the formula milk and the formula milk predominant groups. No significant correlations were found between HBD-2 levels and numbers of *Bifidobacterium, Lactobacillus*, or *Klebsiella* counts (Pearson correlation test, p = 0.513, r = 0.101; p = 0.727, r = 0.054; and p = 0.386, r = 0.134; respectively).

**Table 3.** Intestinal microbiota count in each group

**Type of bacteria**	**Breast milk only (n = 11)**	**Breast milk predominant (n = 11)**	**Formula milk predominant (n = 12)**	**Formula milk only (n = 10)**	**p-value**
*Bifidobacterium* (Mean)	4.68±0.26	4.98±0.29	4.57±0.17	4.93±0.22	0.561
*Bifidobacterium* to total bacteria ratio (%)	60.89±3.94	62.12±3.95	57.09±2.50	59.99±3.03	0.739
*Lactobacillus* (Mean) (%)	4.01±0.23	3.49±0.19	3.59±0.23	3.92±0.27	0.327
*Lactobacillus* to total bacteria ratio	52.07±3.11	43.44±2.14	45.19±3.81	47.69±3.44	0.267
*Klebsiella* (Mean)	4.61±0.19	4.93±0.16	5.12±0.14	5.03±0.12	0.134
*Klebsiella* to total bacteria ratio (%)	59.30±1.42	61.10±1.11	64.07±2.83	61.05±1.37	0.342
Total bacteria	7.78±0.29	8.06±0.16	8.07±0.20	8.23±0.07	0.467

Quantity of specific intestinal microbiota presented as mean±SEM. Bacterial count presented as Loq quantity (copy number DNA /mL feces). One-way ANOVA was assumed as significant at p < 0.05.

## DISCUSSION

### Baseline characteristics.

Our data showed that no significant differences among groups in almost all subject characteristics, i.e. based on sex, age, gestational age, birth weight, body length, head circumference, mode of labor, body temperature, mother's characteristics, and some neonate characteristics, i.e. incidence of asphyxia, sepsis, and oral hygiene problems. However, there were significant differences in ventilator utilization, incidence of PDA, and the usage of a breast milk donor. Furthermore, there was no significant difference of inflammatory markers among groups.

PDA is a common congenital defect found in preterm neonates (20). Normally, the constriction of the ductus arteriosus occurs just after birth and functional closing of the ductus arteriosus occurred at 72 hours of age in term infants (21). Our data showed that PDA incidence was significantly higher in the breast milk only group. However, as long as the PDA did not cause significant hemodynamic problems, the incidence of PDA in the breast milk only group would not disproportionately bias results of this study.

Use of breast milk donors was more common in the formula milk predominant group. Previous studies demonstrated that breast milk contains abundant hBD-2, which has antimicrobial activity - particularly toward pathogenic bacteria (22). However, in our study, the administration of breast milk in the formula milk predominant group was not more frequent compared with the breast milk predominant or breast milk only group thereby this variable will not affect the result of study.

### Human β-defensin 2 levels.

This study showed that hBD-2 levels in the formula milk predominant group were significantly higher compared with the breast milk only group. Moreover, hBD-2 levels were significantly correlated with feeding pattern. This finding suggests that there was a lower inflammatory response in the breast milk only group compared with the formula milk predominant group. The preventative effects of breast milk against some inflammatory intestinal diseases, such as necrotizing enterocolitis, have been investigated (23). The clinical impact of breast milk in that case was correlated with antimicrobial peptides (AMPs) present in the breast milk. Human β-defensins are evolved antimicrobial peptides (AMPs) and are an important part of the innate immune system in the mucosa of the gastrointestinal tract (24).

Thirty-three genes encoding AMPs like human β-defensins (hBD) have been identified in human (10). Of these, there are several types of hBD which have been further investigated, namely hBD-1, hBD-2, and hBD-3 (10, 22). An hBD-2 mRNA was minimally expressed in the gingival keratinocytes, stomach, small intestines, colon, and also mammary gland (22, 25–26) and was found to have two main functions: (1) first-line defense mechanism against pathogens; (2) assisting in the establishment of normal flora on the surface of the body (10). hBD-2 could be upregulated by specific pro-inflammatory (e.g. IL-1α, IL-1β, TNF-α) and microbial molecules through multiple signaling pathways, such as NF-κB (27–28). Based on previous studies, it could be assumed that higher hBD-2 levels in the formula milk predominant group could be caused by an inflammatory response of the intestinal mucosa and were not altered by the abundant content of this AMP in the breast milk.

### Intestinal microbiota pattern.

Statistical analyses found no significant differences in the total number of *Bifidobacterium, Lactobacillus* and *Klebsiella* nor in their respective ratios to total bacteria among groups. Despite this lack of statistical significance, the quantity of *Lactobacillus* was found to be highest in the breast milk only group. The *Bifidobacterium* count was higher in breast milk only and breast milk predominant groups, whereas the *Klebsiella* count was higher in the formula milk only and formula milk predominant groups.

Previous evidence shows that delayed establishment of non-pathogenic intestinal microbiota (mainly *Lactobacillus* and *Bifidobacterium*) in preterm infants can affect long term health (29); probiotic and breast milk administration are known to have a positive impact on the development of *Lactobacillus* and *Bifidobacterium* (30–31). Breast-fed infants possess a higher composition of *Bifidobacterium* and lactic acid bacteria (LAB), while formula milk fed infants have a more diverse microbial pattern that includes *Bifidobacterium, Bacteroides, Clostridium, Streptococcus* and higher numbers of facultative anaerobic bacteria, such as *Staphylococcus, Streptococcus* and *Enterobacteriaceae* (32–33).

A previous study has shown that *Lactobacillus* constituencies were significantly dependent on probiotic administration and the number of *Lactobacillus* would decrease as probiotic usage stopped (30). Moreover, *Bifidobacterium* was selectively developed in breast fed infants because of the high number of bifidogenic factors, such as oligosaccharides, in human milk. These bifidogenic factors are difficult for infants to digest, and so become a substrate for fermentation in the distal gut where they trigger the growth of *Bifidobacterium* (34). Donor human milk also influences the composition of gut microbiota and is characterized by a higher number of *Bifidobacterium*, mimicking the mother's own milk and having a different pattern to formula fed infants (35).

PCR has been used to show that bands corresponding to *E. coli, Enterococcus sp.*, and *Klebsiella pneumoniae* were the most common gut microbiota in preterm infants (29) and formula-fed infants (35). Colonization of pathogenic bacteria, which are more dominant in preterm infants, could trigger intestinal and systemic inflammatory disease (36). As there was significant usage of ventilators in the breast milk only group in the current study, the pattern of intestinal microbiota might have been influenced by the oxygen fraction, as was reported in previous studies (8, 37).

## CONCLUSION

hBD-2 levels were significantly higher in the formula milk predominant group compared with the breast milk only group. Gut microbiota patterns showed that *Bifidobacterium* and *Lactobacillus* were higher in the breast milk group, while *Klebsiella* was higher in the formula milk only group, but these differences were not statistically significant.
